# Imeglimin Exhibits Novel Anti-Inflammatory Effects on High-Glucose-Stimulated Mouse Microglia through ULK1-Mediated Suppression of the TXNIP–NLRP3 Axis

**DOI:** 10.3390/cells13030284

**Published:** 2024-02-05

**Authors:** Hisashi Kato, Kaori Iwashita, Masayo Iwasa, Sayaka Kato, Hajime Yamakage, Takayoshi Suganami, Masashi Tanaka, Noriko Satoh-Asahara

**Affiliations:** 1 Department of Endocrinology, Metabolism and Hypertension Research, Clinical Research Institute, NHO Kyoto Medical Center, Kyoto 612-8555, Japan; hkato@mail.doshisha.ac.jp (H.K.);; 2 Department of Endocrinology and Metabolism, Graduate School of Medical Science, Kyoto Prefectural University of Medicine, Kyoto 602-8566, Japan; 3 Department of Molecular Medicine and Metabolism, Research Institute of Environmental Medicine, Nagoya University, Nagoya 464-8601, Japan; 4 Department of Immunometabolism, Nagoya University Graduate School of Medicine, Nagoya 464-8601, Japan; 5 Institute of Nano-Life-Systems, Institutes of Innovation for Future Society, Nagoya University, Nagoya 464-8601, Japan; 6 Center for One Medicine Innovative Translational Research (COMIT), Nagoya University, Nagoya 464-8601, Japan; 7 Department of Rehabilitation, Health Science University, Minamitsuru-gun 401-0380, Japan; 8 Department of Metabolic Syndrome and Nutritional Science, Research Institute of Environmental Medicine, Nagoya University, Nagoya 466-8550, Japan

**Keywords:** imeglimin, microglia, ULK1, hyperglycemia, neuroinflammation, type 2 diabetes mellitus

## Abstract

Type 2 diabetes mellitus (T2DM) is an epidemiological risk factor for dementia and has been implicated in multifactorial pathologies, including neuroinflammation. In the present study, we aimed to elucidate the potential anti-inflammatory effects of imeglimin, a novel antidiabetic agent, on high-glucose (HG)-stimulated microglia. Mouse microglial BV2 cells were stimulated with HG in the presence or absence of imeglimin. We examined the effects of imeglimin on the levels of proinflammatory cytokines, intracellular reactive oxygen species (ROS), mitochondrial integrity, and components related to the inflammasome or autophagy pathways in these cells. Our results showed that imeglimin suppressed the HG-induced production of interleukin-1beta (IL-1β) by reducing the intracellular ROS levels, ameliorating mitochondrial dysfunction, and inhibiting the activation of the thioredoxin-interacting protein (TXNIP)–NOD-like receptor family pyrin domain containing 3 (NLRP3) axis. Moreover, the inhibitory effects of imeglimin on the TXNIP–NLRP3 axis depended on the imeglimin-induced activation of ULK1, which also exhibited novel anti-inflammatory effects without autophagy induction. These findings suggest that imeglimin exerted novel suppressive effects on HG-stimulated microglia through the ULK1–TXNIP–NLRP3 axis, and may, thereby, contribute to the development of innovative strategies to prevent T2DM-associated cognitive impairment.

## 1. Introduction

Type 2 diabetes mellitus (T2DM) is a major public health concern with an increasing global prevalence [1]. Furthermore, T2DM is an epidemiological risk factor for dementia, a serious health issue that continues to expand worldwide [2,3,4]. Therefore, there is an urgent need to elucidate the underlying mechanisms and develop novel preventive and therapeutic strategies for DM-associated dementia.

Multifactorial pathways have been implicated in the pathogenesis of T2DM-associated cognitive impairment, such as oxidative stress, neuroinflammation, cerebrovascular damage, and central insulin resistance [4,5,6]. Although the underlying mechanistic details have not been fully elucidated, the production of reactive oxygen species (ROS) in diabetes is involved in hyperglycemia-induced mitochondrial dysfunction [7,8]. Hyperglycemia also plays a pathological role in neuroinflammation by activating microglia and resident immune cells in the brain [8,9]. Reportedly, the administration of high levels of glucose triggers microglial inflammatory responses and results in the production of proinflammatory cytokines and damage-associated molecular patterns (DAMPs) via ROS and multiple signaling pathways, including nuclear factor-kappa B (NF-κB) and mitogen-activated protein kinase (MAPK) pathways [10,11,12]. Furthermore, previous studies have demonstrated the roles of NOD-like receptor family pyrin domain containing 3 (NLRP3) inflammasome activation in the production of interleukin-1beta (IL-1β) in microglia under high glucose (HG) conditions [10,13]. The NLRP3 inflammasome comprises the sensor molecule NLRP3, the adaptor protein apoptosis-associated speck-like protein containing a caspase recruitment domain (ASC) and pro-caspase-1; its activation leads to IL-1β production and triggers a highly inflammatory type of programmed cell death termed pyroptosis [14]. In addition, we recently demonstrated a potential role for thioredoxin-interacting protein (TXNIP), a regulator of oxidative stress, in IL-1β production that is mediated by the activation of the TXNIP–NLRP3 axis in HG-stimulated microglia [15]. Since neuroinflammation is implicated in the exacerbation of dementia-related brain pathologies [16], the suppression of microglial activation would be effective for ameliorating T2DM-associated cognitive impairment.

Recent preclinical studies in mouse models of diabetes have reported the beneficial effects of certain antidiabetic drugs, such as dipeptidyl peptidase-4 (DPP-4) inhibitors and metformin, on neuroinflammation and cognitive impairment [17,18,19]. A meta-analysis of antidiabetic agents for dementia risk also demonstrated that patients with T2DM treated with DPP-4 inhibitors had the lowest risk of dementia, followed by those treated with metformin and thiazolidinediones [20]. These findings suggest that some medications for T2DM may possess preventive and/or therapeutic potential against cognitive impairment. However, the therapeutic efficacy of antidiabetic agents against cognitive impairment in humans has not been established [21].

Imeglimin is a novel oral antidiabetic agent for the treatment of T2DM that targets mitochondrial bioenergetics, enabling the modulation of glucose-stimulated insulin secretion by improving β-cell function [22,23,24]. Imeglimin improves hyperglycemia through a variety of mechanisms, such as the improvement of mitochondrial function by regulating the activity of the mitochondrial respiratory chain complex and subsequent suppression of ROS production [25,26]. Importantly, a recent pharmacokinetic study in rats reported that orally administered imeglimin was detected in the brain, indicating that imeglimin could cross the blood–brain barrier (BBB) [27]. Accordingly, these findings suggest the novel suppressive effects of imeglimin on ROS production and microglial activation under diabetic conditions, although the mechanisms by which imeglimin exerts its effects on these cells remain unclear. In this study, we focused on the in vitro action of imeglimin and examined its effects on HG-induced microglial inflammatory responses.

## 2. Materials and Methods

### 2.1. Cell Culture and Treatment

The mouse microglia cell line BV2 (donated by Drs. Yoneda (Kanazawa University, Kanazawa, Japan) and Hinoi (Gifu Pharmaceutical University, Gifu, Japan)) was cultured in Dulbecco’s Modified Eagle’s Medium with low glucose (5.6 mmol/L glucose, Fujifilm Wako, Osaka, Japan) containing 10% heat-inactivated fetal bovine serum (BioWest, Bradenton, FL, USA) and 1% penicillin and streptomycin (Fujifilm Wako). Cells were maintained in a humidified atmosphere containing 5% CO_2_ at 37 °C. Using an in vitro hyperglycemic model, HG-induced inflammatory responses were examined as previously reported [11], with some modifications. BV2 cells were seeded at 2 × 10^5^ and 4 × 10^5^ cells/well in 24- and 12-well plates, respectively. After incubation for 24 h, the cells were pretreated with imeglimin at a final concentration of 500 μM or the vehicle control for 30 min and cultured for an additional 24 h with the same culture medium containing 5.6 mM glucose (low glucose (LG)), or with glucose at a final concentration of 75 mM (high glucose (HG)), with or without imeglimin. Imeglimin (provided by Sumitomo Pharma Co. Ltd., Osaka, Japan) was first dissolved in distilled H_2_O, sterilized by filtration, to prepare a stock solution (100 mM) and then diluted to the concentrations of interest using the low LG culture medium. The vehicle control for imeglimin treatment contained the same volume of sterilized distilled H_2_O. Culture supernatant was collected to measure the proinflammatory cytokines (IL-1β and tumor necrosis factor-α [TNF-α]), and the cells were then washed, harvested, and further analyzed.

### 2.2. Cytotoxicity Assays

The cytotoxicity of imeglimin in BV2 cells was determined using a Cell Counting Kit-8 (CCK-8; Dojindo Laboratories (Dojindo), Kumamoto, Japan) according to the manufacturer’s instructions. Briefly, BV2 cells were seeded in 96-well plates at a density of 5 × 10^3^ cells/well, followed by treatment with different concentrations of imeglimin or vehicle control. After incubating the BV2 cells with imeglimin or the vehicle for 24 h in a CO_2_ incubator, 10 μL of CCK-8 (tetrazolium salt) solution was added to each well and incubated for 2 h. The optical density (OD) at 450 nm was measured using a microplate reader to determine the amount of orange-colored formazan dye that was generated through dehydrogenase activity in viable cells. The results are shown as the percentage cell viability compared with the 0 μM imeglimin group.

### 2.3. Total RNA Extraction and Quantitative RT-PCR

Total RNA was extracted from the BV2 cells using an RNeasy Mini Kit (QIAGEN, Germantown, MD, USA), and first-strand cDNA was synthesized using a High-Capacity RNA-to-cDNA Kit (Applied Biosystems, Waltham, MA, USA) according to the manufacturer’s instructions. The expression levels of the genes of interest were determined through quantitative reverse transcription polymerase chain reaction (RT-PCR) using the Power SYBR Green PCR Master Mix (Applied Biosystems) and StepOnePlusTM Real-Time PCR System (Applied Biosystems). Relative changes in gene expression were determined using the 2^−ΔΔCt^ method. All quantitative RT-PCR results were normalized to 18S ribosomal RNA expression. The primer sequences used in this study are listed in Supplementary Table S1.

### 2.4. Cytokine Measurements

Proinflammatory cytokines IL-1β and TNF-α in the culture supernatant were quantified using the Mouse IL-1β and TNF-alpha ELISA kits (Proteintech, Rosemont, IL, USA), according to the manufacturer’s instructions. The OD at 450 nm was measured using a microplate reader. The concentration of each proinflammatory cytokine was calculated using a calibration curve of known concentrations of the respective proinflammatory cytokines.

### 2.5. Western Blot Analysis

Western blot analysis was performed as previously described, with minor modifications [28]. BV2 cells were washed twice with phosphate-buffered saline and homogenized in an ice-cold radioimmunoprecipitation assay lysis buffer (20 mM HEPES, pH 7.5, 1% NP-40, 0.1% SDS, 0.5% deoxycholic acid, and 150 mM sodium chloride) supplemented with HaltTM Protease and Phosphatase Inhibitor Cocktail (Thermo Scientific, Waltham, MA, USA). The homogenate was incubated on ice for 15 min and centrifuged for 20 min at 14,000× *g* at 4 °C. Samples were frozen and stored at −80 °C until analysis. Equal amounts of cellular protein were loaded onto each well, resolved using sodium dodecyl-sulfate (SDS)-polyacrylamide gel electrophoresis, and transferred to polyvinylidene fluoride membranes (ATTO, Tokyo, Japan). The membrane was blocked for 5 min with Bullet Blocking One (Nacalai Tesque, Kyoto, Japan), or for 60 min in TBS-T containing 5% BSA, followed by overnight incubation with 1:1000-diluted specific antibodies (Supplementary Table S2) at 4 °C. Subsequently, the membrane was labeled for 60 min with 1: 3000-diluted HRP-conjugated anti-rabbit or anti-mouse IgG secondary antibodies (Cell Signaling Technology, Danvers, MA, USA) and detected using EzWestLumi plus (ATTO). Each band was imaged using the ChemiDoc XRS Plus imaging system (Bio-Rad, Hercules, CA, USA), and protein levels were quantified by analyzing the band intensities using ImageJ software (version 1.53k, NIH, Bethesda, MD, USA).

### 2.6. Mitochondrial ROS Measurement

The levels of intracellular ROS were evaluated using a Highly Sensitive DCFH-DA-ROS assay kit (Dojindo) according to the manufacturer’s instructions. Concisely, BV2 cells were seeded in a μ-Slide 8 Well (ibidi GmbH, Gräfelfing, Germany) at a density of 5 × 10^3^ cells/well and cultured with imeglimin or vehicle control under LG or HG conditions. After washing twice with Hanks’ balanced salt solution (HBSS), the cells were treated with the highly sensitive DCFH-DA working solution for 30 min in a CO_2_ incubator. Cells were then washed with HBSS, and fluorescence signals were observed using a fluorescence microscope (ZEISS, Land Baden-Württemberg, Germany). The fluorescence intensity of ROS in each group was quantified using ImageJ software (NIH).

### 2.7. Mitochondrial Membrane Potential Measurement

The JC-1 MitoMP detection kit (Dojindo) was used to determine the mitochondrial membrane potential (MMP), according to the manufacturer’s instructions. Briefly, BV2 cells were seeded in a μ-Slide 8 Well (ibidi GmbH) at a density of 5 × 10^3^ cells/well and cultured as described in Section 2.6. Cells were incubated with JC-1 working solution (4 μmol/L) for 30 min in a CO_2_ incubator, subsequently washed with HBSS, and observed using a fluorescence microscope (ZEISS). JC-1 indicates MMP, and a decrease in the red/green fluorescence intensity ratio reflects mitochondrial depolarization. The relative red/green fluorescence intensity ratio of JC-1 in each group was determined using ImageJ software (NIH).

### 2.8. siRNA Transfection

BV2 cells (50–70% confluent) were transfected for 24 h in 24-well plates with 10 pmol/L small interfering RNA (siRNA) targeting ULK1 (MISSION siRNA; Sigma-Aldrich, St. Louis, MO, USA) using Lipofectamine RNAiMAX reagent (Thermo Scientific) in accordance with the manufacturer’s instructions. The MISSION siRNA Universal Negative Control (Sigma-Aldrich) was used as a control. After transfection, the cells were treated with imeglimin or vehicle for 30 min under LG conditions, followed by incubation with LG or HG for 24 h in the presence of imeglimin or vehicle as described above. The cells were harvested and used for mRNA or protein quantification using quantitative RT-PCR or Western blot analyses.

### 2.9. Statistical Analysis

All data are presented as the mean ± SEM. Means were compared between groups using Student’s *t*-test or one-way analysis of variance (ANOVA) with Tukey’s post hoc test for pairwise comparisons using GraphPad Prism version 9 (GraphPad Software, San Diego, CA, USA). *p* < 0.05 was considered statistically significant.

## 3. Results

### 3.1. Imeglimin Exerted Anti-Inflammatory Effects on High-Glucose-Activated BV2 Microglia

**Figure 1:**
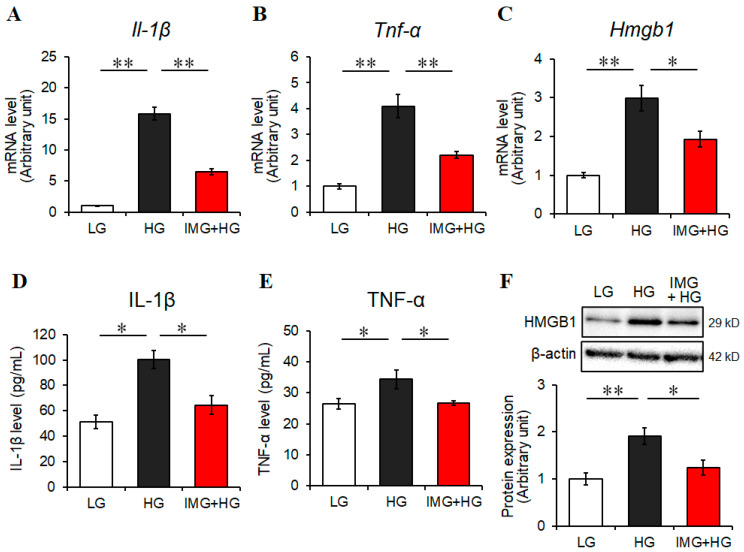
Effects of imeglimin on inflammatory mediators in high glucose (HG)-stimulated BV2 cells. Cells were pretreated with 500 μM imeglimin or the vehicle control for 30 min and then incubated for 24 h under low glucose or HG conditions. Expression levels of genes related to inflammatory mediators *Il-1β* (**A**), *Tnf-α* (**B**), and *Hmgb1* (**C**) were analyzed using real-time RT-PCR with 18S rRNA used as an internal control. The amounts of proinflammatory cytokines IL-1β (**D**) and TNF-α (**E**) in the culture supernatant were quantified using ELISA. HMGB1 protein levels in the microglia were determined through Western blot (**upper panel**: representative images) and densitometry (**lower panel**) (**F**). The amount of HMGB1 was normalized to that of β-actin. Expression levels are displayed relative to vehicle controls (1.0) (**A**–**C**,**F**). Data are presented as the mean ± SEM from three independent experiments (*n* = 3). * *p* < 0.05, ** *p* < 0.01, SEM: standard error of the mean. LG: low glucose, HG: high glucose, IMG: imeglimin, IL-1β: interleukin-1β, TNF-α: tumor necrosis factor alpha, HMGB1: high mobility group box 1.

First, we tested for cytotoxic effects of imeglimin on BV2 cells. The cells were treated with various concentrations (0–1000 μM) of imeglimin for 24 h in a 5.6 mM LG medium, and we found that imeglimin, up to 1000 μM, exhibited no significant cytotoxicity on BV2 microglia under our experimental conditions (Supplementary Figure S1). To examine the potential neuroprotective effects of imeglimin, we investigated whether imeglimin influenced the levels of inflammatory mediators in HG-activated BV2 cells. Our results revealed that the gene expression levels of the proinflammatory cytokines *Il-1β* and *Tnf-α* and the inflammatory mediator *Hmgb1* [29] were significantly upregulated in BV2 cells under HG conditions, but these were significantly decreased by imeglimin treatment (500 μM); Figure 1A–C. Similarly, the secretion levels of IL-1β and TNF-α and the protein expression level of HMGB1 were significantly increased under HG conditions, and imeglimin significantly reduced the levels of these proteins in BV2 cells (Figure 1D–F). These results indicate that imeglimin exerts anti-inflammatory effects on HG-stimulated BV2 microglia.

### 3.2. Imeglimin Attenuated ROS Generation, Loss of MMP, Activation of Mitophagy, and Apoptosis Induction in High-Glucose-Activated BV2 Microglia

**Figure 2:**
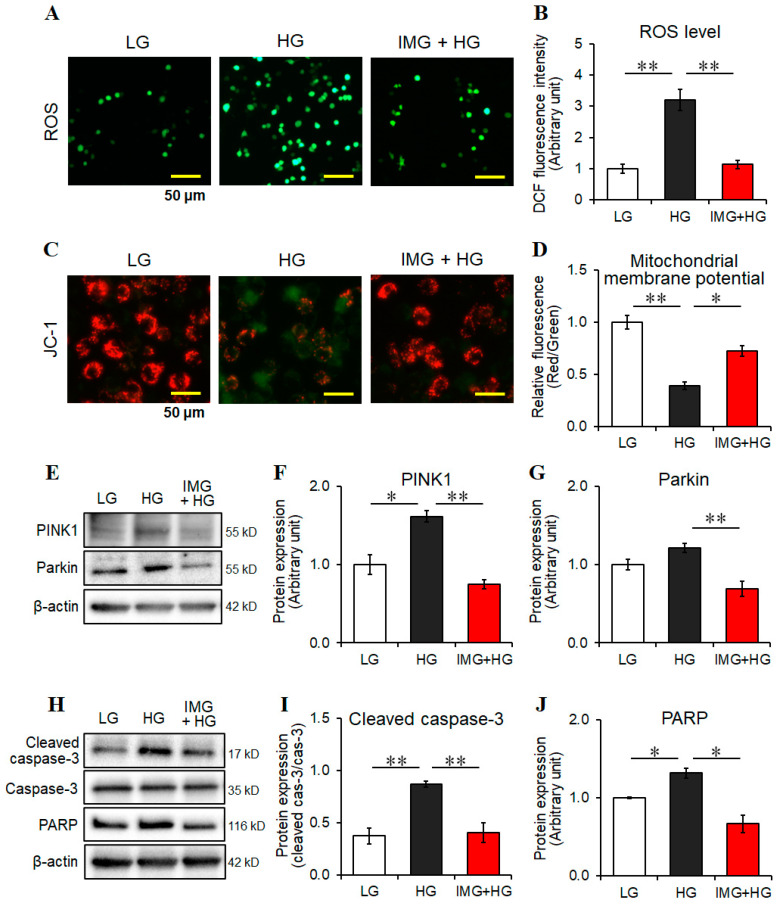
Effects of imeglimin on reactive oxygen species (ROS) generation, mitochondrial membrane potential (MMP), mitophagy, and apoptosis in high glucose (HG)-stimulated BV2 cells. Cells were pretreated with 500 μM imeglimin or the vehicle control for 30 min and then incubated for 24 h under low glucose or HG conditions. Intercellular ROS levels were examined using the DCFH-DA assay; fluorescence images (**A**) were obtained using a fluorescence microscope (scale bar, 50 μm), and the ROS levels (**B**) were quantified by measuring the fluorescence intensities. MMP levels were examined using the JC-1 assay; fluorescence images (**C**) were acquired (scale bar, 50 μm), and the MMP levels (**D**) were quantified by measuring the fluorescence intensities. Mitophagy components PINK1 and Parkin were analyzed using Western blot (**E**) and densitometry (**F**,**G**). Apoptosis-related proteins, cleaved cas-3 and PARP, were examined using Western blot (**H**) and densitometry (**I**,**J**). The amount of protein of interest was normalized to that of β-actin (**F**,**G**,**J**) or cas-3 (**I**). Fold changes are displayed relative to vehicle controls (1.0) (**B**,**D**,**F**,**G**,**J**). Data are presented as the mean ± SEM from three independent experiments (*n* = 3). * *p* < 0.05, ** *p* < 0.01, SEM: standard error of the mean. LG: low glucose, HG: high glucose, IMG: imeglimin, PINK1: phosphatase and tensin homolog (PTEN)-induced putative kinase 1, cleaved cas-3: cleaved caspase-3, cas-3: caspase-3, PARP: poly(ADP-ribose) polymerase, 2′,7′-DCFH-DA: dichloro-dihydro-fluorescein diacetate.

HG-induced intracellular ROS has been shown to trigger an inflammatory response and may be related to mitochondrial dysfunction in microglial cells [8,12,30]. Therefore, we investigated whether the anti-inflammatory effects of imeglimin on HG-stimulated microglia involve intercellular ROS levels and mitochondrial integrity. The HG environment significantly increased intercellular ROS levels, which were significantly reduced by imeglimin treatment (Figure 2A,B). In addition, HG treatment significantly reduced the ratio of red to green JC-1 fluorescence, suggesting a decrease in MMP, which reflects mitochondrial dysfunction (Figure 2C,D). In contrast, this decrease in MMP levels was significantly inhibited by imeglimin treatment (Figure 2C,D). These results suggest that imeglimin reduces intracellular ROS levels and, subsequently, prevents mitochondrial dysfunction, thereby exerting anti-inflammatory effects on BV2 microglia under HG conditions.

Mitophagy is a process that selectively degrades damaged mitochondria and is essential for cellular health [31]. A previous study reported the suppressive roles of PINK1/Parkin-mediated mitophagy in neuroinflammation [32]; therefore, we examined whether imeglimin induces mitophagy to inhibit microglial activation under HG conditions. We observed that PINK1 levels were significantly increased by HG stimulation, and that imeglimin significantly reduced PINK1 levels in HG-activated BV2 cells (Figure 2E,F). Imeglimin also reduced Parkin levels under HG conditions, whereas HG treatment did not significantly affect the levels of Parkin in microglia (Figure 2E,G). Furthermore, the protein levels of the apoptosis-related factors, cleaved caspase-3 and PARP, were significantly increased under HG conditions, and imeglimin treatment downregulated these proteins in microglia (Figure 2H–J). These results revealed that mitophagy and apoptosis were not significantly induced by imeglimin, potentially due to the alleviation of oxidative stress and the prevention of mitochondrial dysfunction, thereby suggesting that imeglimin plays no significant role in mitophagy-mediated anti-inflammatory actions in microglia under HG conditions.

### 3.3. Imeglimin Suppressed Activation of the TXNIP–NLRP3 Inflammasome Pathway in High-Glucose-Activated BV2 Microglia

**Figure 3:**
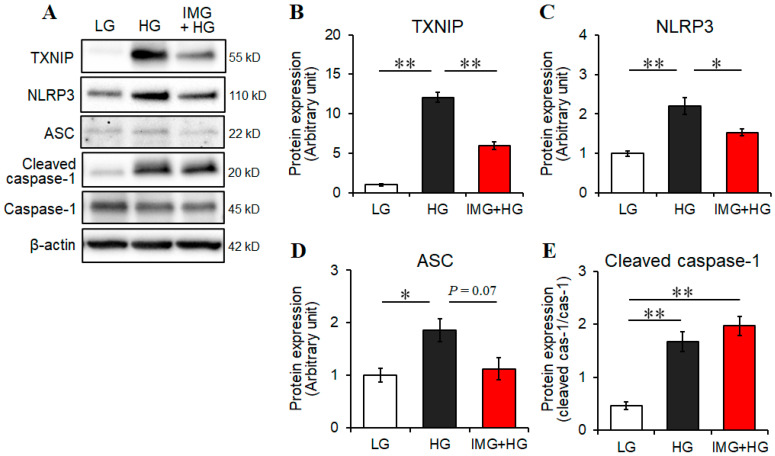
Effects of imeglimin on the activation of the TXNIP–NLRP3 axis in high glucose (HG)-stimulated BV2 cells. Cells were pretreated with 500 μM imeglimin or the vehicle control for 30 min and then incubated for 24 h under low glucose or HG conditions. Proteins of interest were analyzed using Western blot (**A**). The levels of TXNIP (**B**), NLRP3 (**C**), or ASC (**D**) relative to β-actin and the levels of cleaved cas-1 (**E**) relative to cas-1 were determined using densitometry. Expression levels are displayed relative to vehicle controls (1.0) (**B**–**D**). Data are presented as the mean ± SEM from three independent experiments (*n* = 3). * *p* < 0.05, ** *p* < 0.01, SEM: standard error of the mean. LG: low glucose, HG: high glucose, IMG: imeglimin, TXNIP: thioredoxin-interacting protein, NLRP3: NOD-like receptor family pyrin domain containing 3, ASC: apoptosis-associated speck-like protein containing a caspase recruitment domain, cleaved cas-1: cleaved caspase-1, cas-1: caspase-1.

We recently reported that HG-induced ROS triggers inflammatory responses by activating the TXNIP–NLRP3 axis in microglia [15]. Therefore, we addressed the possibility that the anti-inflammatory effects of imeglimin are related to the inhibition of the TXNIP–NLRP3 axis in BV2 cells under HG conditions. Compared with LG conditions, the levels of TXNIP, NLRP3, and ASC and the ratio of cleaved caspase-1/pro-caspase-1 were significantly elevated under HG conditions (Figure 3A–E). Moreover, we observed that imeglimin treatment significantly reduced the levels of TXNIP and NLRP3 and almost significantly reduced ASC levels, whereas the ratio of cleaved caspase-1/pro-caspase-1 was not significantly affected by imeglimin under HG conditions (Figure 3A–E). These results suggest that imeglimin exerts anti-inflammatory effects on HG-activated BV2 microglia by attenuating the TXNIP–NLRP3 axis in these cells.

### 3.4. Imeglimin Had No Significant Effect on Autophagy Induction in High-Glucose-Activated BV2 Microglia

**Figure 4:**
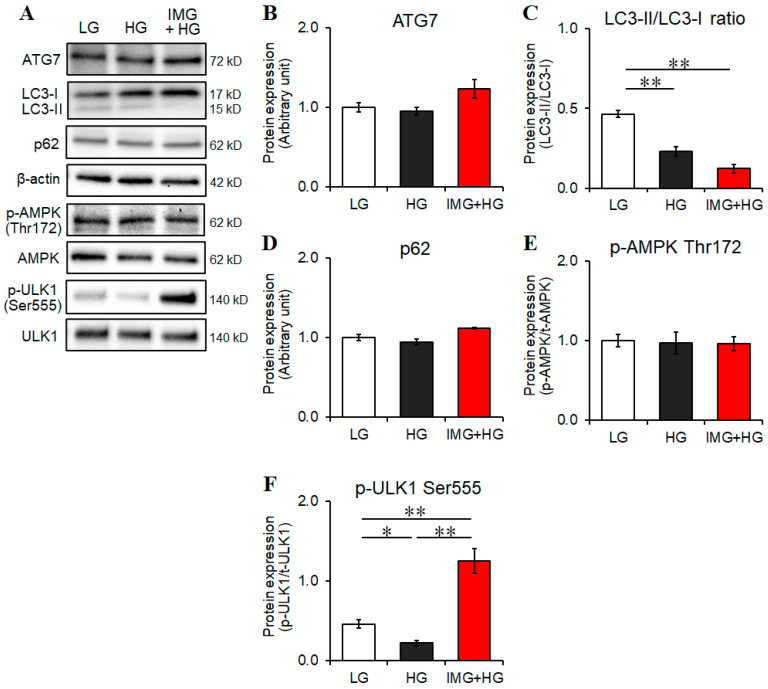
Effects of imeglimin on autophagy component levels in high glucose (HG)-stimulated BV2 cells. Cells were pretreated with 500 μM imeglimin or the vehicle control for 30 min and then incubated for 24 h under low glucose or HG conditions. Expression levels of the proteins of interest were analyzed using Western blot (**A**) and densitometry (**B**–**F**); the protein levels of (**B**) ATG7 relative to β-actin; (**C**) LC3-II relative to LC3-I; (**D**) p62 relative to β-actin; (**E**) p-AMPKα relative to total AMPKα; (**F**) p-ULK1 relative to total ULK1. Protein levels are displayed relative to vehicle controls (1.0) (**B**,**D**). Data are presented as the mean ± SEM from three independent experiments (*n* = 3). * *p* < 0.05, ** *p* < 0.01, SEM: standard error of the mean. LG: low glucose, HG: high glucose, IMG: imeglimin, ATG: autophagy-related, LC3: microtubule-associated protein light chain 3, AMPK: adenosine monophosphate-activated protein kinase, t-AMPK: total AMPK, ULK1: UNC-51 like autophagy activating kinase 1, t-ULK1: total ULK1.

A recent study reported that autophagy, a catabolic process that degrades damaged organelles and protein aggregates, suppresses the production of proinflammatory cytokines in activated microglia [33]. Moreover, a mouse liver study demonstrated that autophagy inhibited the ROS-mediated activation of the TXNIP–NLRP3 axis and, consequently, reduced the levels of inflammatory mediators [34]. Therefore, we investigated whether imeglimin induces autophagy to suppress the TXNIP–NLRP3 axis in microglia under HG conditions. Our results revealed that the significant upregulation of ATG7 protein and the ratio of LC3-II/LC3-I, which are indicators of autophagy induction [32], were not observed following HG or imeglimin treatment (Figure 4A–C). Furthermore, HG or imeglimin treatment did not significantly downregulate p62 (Figure 4D), another indicator of autophagy induction [32]. These results suggest that imeglimin and HG did not significantly induce autophagy in microglia.

In line with these findings, HG or imeglimin treatment did not significantly activate AMPK, which is involved in autophagy initiation [35] (Figure 4E). However, we observed notable changes in the activation state of ULK1, which regulates autophagy induction, when phosphorylated by AMPK [35]. ULK1 is activated by phosphorylation at Ser555 and positively regulates autophagy induction [36]. Despite no significant activation of AMPK (Figure 4E), the phosphorylation levels of ULK1 were reduced under HG conditions and activated by imeglimin treatment in BV2 cells (Figure 4F). These results suggest that HG or imeglimin treatment affected the activity of the autophagy-related protein ULK1 without AMPK activation, whereas autophagy was not significantly induced under these conditions.

### 3.5. Imeglimin Exhibited ULK1-Dependent Anti-Inflammatory Effects on High-Glucose-Stimulated BV2 Microglia

**Figure 5:**
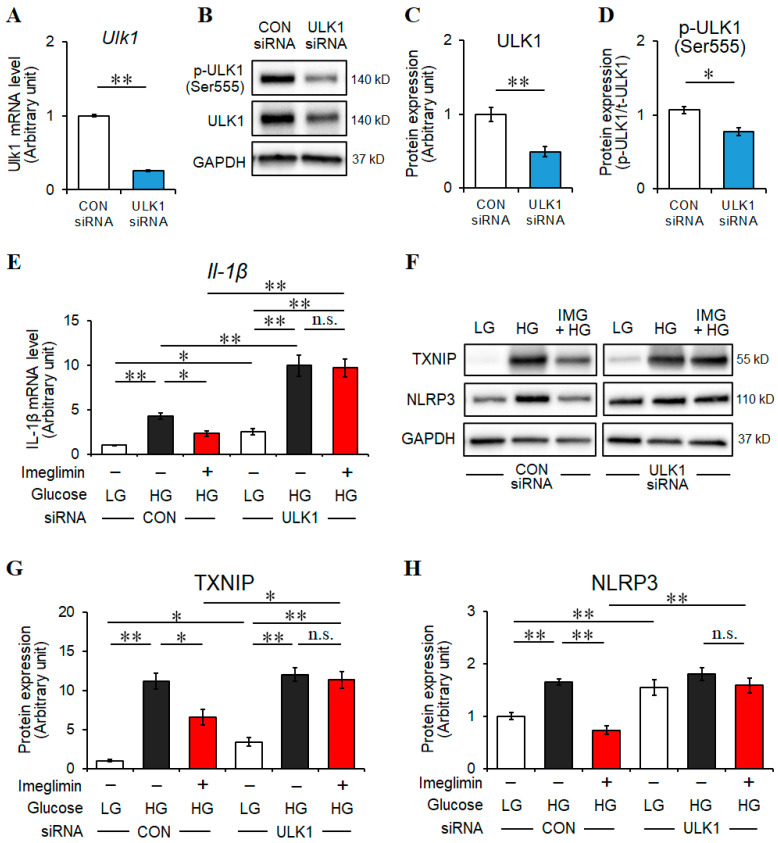
Effects of imeglimin on inflammatory responses of high glucose (HG)-stimulated BV2 cells with or without ULK1 silencing. BV2 cells were transfected with ULK1 siRNA or control siRNA for 24 h and then subjected to treatment with 500 μM imeglimin or the vehicle control for 30 min, followed by incubation for 24 h under low glucose or HG conditions in the continued presence of imeglimin or the vehicle control. *Ulk1* mRNA levels 24 h after siRNA transfection were examined using quantitative RT-PCR and normalized to those of 18S rRNA (**A**). Protein levels of interest were determined using Western blot (**B**) and densitometry (**C**,**D**); (**C**) the amount of ULK1 relative to GAPDH; (**D**) the amount of p-ULK1 relative to total ULK1. *Il-1β* mRNA levels were examined using quantitative RT-PCR and normalized to those of 18S rRNA (**E**). Protein levels of TXNIP, NLRP3, and GAPDH were determined using Western blot (**F**). The amount of TXNIP (**G**) or NLRP3 (**H**) relative to GAPDH was obtained through densitometry. Fold changes are displayed relative to vehicle controls (1.0) (**A**,**C**,**E**,**G**,**H**). Data are presented as the mean ± SEM from three independent experiments (*n* = 3). * *p* < 0.05, ** *p* < 0.01, n.s., not significant, SEM: standard error of the mean. CON: control, ULK1: UNC-51 like autophagy activating kinase 1, t-ULK1: total ULK1, LG: low glucose, HG: high glucose, IMG: imeglimin, TXNIP: thioredoxin-interacting protein, NLRP3: NOD-like receptor family pyrin domain containing 3, GAPDH: glyceraldehyde-3-phosphate dehydrogenase.

To determine whether ULK1 is involved in the anti-inflammatory effects of imeglimin in HG-stimulated BV2 cells, we investigated the effects of ULK1 silencing on microglia through transfecting control or ULK1 siRNA (Figure 5A–D). If ULK1 is implicated in the anti-inflammatory effects of imeglimin treatment, siRNA targeting ULK1 should attenuate the suppressive effects of imeglimin on IL-1β, TXNIP, and NLRP3 levels.

Unexpectedly, our results revealed that ULK1 siRNA significantly increased the gene expression levels of IL-1β compared with control siRNA in HG-stimulated BV2 microglia (Figure 5E), thereby suggesting that ULK1 potentially suppresses the inflammatory responses in the microglia without imeglimin treatment. We further found that the suppressive effects of imeglimin on IL-1β expression were abolished through ULK1 silencing (Figure 5E), indicating the involvement of ULK1 in the anti-inflammatory effects of imeglimin. Moreover, there was no significant difference in the levels of IL-1β between HG-activated microglia with and without imeglimin treatment when ULK1 expression was silenced (Figure 5E). Therefore, these results suggest that ULK1 exerted novel suppressive effects on IL-1β production and imeglimin further promoted the effects of ULK1 in the microglia under HG conditions.

Similar results were obtained for the levels of TXNIP and NLRP3 (Figure 5F–H), with the exception of no significant difference in NLRP3 levels under LG and HG conditions in microglia transfected with ULK1 siRNA (Figure 5F,H), suggesting the involvement of ULK1 in the anti-inflammatory effects of imeglimin, which suppresses the TXNIP–NLRP3 axis.

We further found that in an LG environment, ULK1 siRNA significantly increased the IL-1β, TXNIP, and NLRP3 levels compared with control siRNA (Figure 5E–H). These results highlight the novel anti-inflammatory role of ULK1 in microglia promoted by imeglimin treatment.

## 4. Discussion

In the present study, we provide the first evidence that imeglimin exerts novel anti-inflammatory effects on HG-activated microglia by suppressing the TXNIP–NLRP3 axis. Furthermore, the suppressive effects of imeglimin on the TXNIP–NLRP3 axis are dependent on ULK1, which also exerts novel autophagy-independent anti-inflammatory effects. Therefore, the mechanism underlying the anti-inflammatory effects of imeglimin could be that imeglimin enhances the ULK1-mediated suppression of the TXNIP–NLRP3 axis, thereby inhibiting microglial activation. These findings demonstrate the novel functional significance of imeglimin in preventing cognitive impairment in diabetes.

ULK1 is involved in initiating autophagy [37], and ULK1-mediated autophagy induction negatively impacts NLRP3 inflammasome activation, thereby suppressing proinflammatory cytokines [38]. However, no studies elucidating the autophagy-independent implications of ULK1 in anti-inflammatory processes or the potential anti-inflammatory effects of imeglimin have been reported. We found that the TXNIP, NLRP3, and IL-1β levels were significantly increased by ULK1 silencing even under an LG environment; therefore, ULK1 could suppress these inflammatory pathways that would be activated by the ROS produced under physiological conditions. Moreover, the suppressive effects of ULK1 were more significant under HG conditions because HG results in higher levels of intracellular ROS production and triggers inflammatory responses. Herein, imeglimin activated ULK1 which subsequently inhibited the TXNIP–NLRP3 axis and suppressed IL-1β production, without autophagy induction. Accordingly, these results suggest that imeglimin augments the novel anti-inflammatory effects of ULK1 on microglia under HG conditions.

ULK1 is activated by AMPK-mediated phosphorylation at Ser555 [35,36]. The present study showed that imeglimin significantly activated ULK1, whereas the activation of AMPK was not significantly affected by imeglimin treatment. Therefore, imeglimin activated ULK1 through pathways other than via AMPK, further suggesting the existence of novel mechanisms of ULK1 activation implicated in anti-inflammatory responses. Moreover, as autophagy was not induced by imeglimin, despite ULK1 activation, novel ULK1-mediated cellular processes exist for anti-inflammatory actions stimulated by imeglimin. Supporting these possibilities, recent studies have unveiled the non-autophagic functions of ULK1 in various cellular processes [39]. Further studies are required to elucidate the mechanistic details underlying the imeglimin-stimulated ULK1–TXNIP–NLRP3 axis as well as to identify the target molecules of imeglimin and ULK1.

There are some limitations in the present study. First, this is an in vitro study to elucidate the anti-inflammatory potentials and the underlying mechanisms of imeglimin. Future studies using mice models of T2DM and/or dementia, as well as clinical studies, are warranted to clarify the in vivo effects of imeglimin. Second, the present study was performed using BV2 microglial cells. Additional studies using another cell line or primary microglia would corroborate the findings of this study. Third, autophagy flux [40] was not investigated, although the effects of imeglimin on autophagy-related markers were examined. The analysis of autophagy flux with and without ULK1 silencing would be valuable for further understanding of the mechanisms underlying ULK1-related anti-inflammatory pathways. Finally, additional studies are required to explore the effects of imeglimin on inflammation-related signaling pathways, such as NF-κB signaling, p38, and SAPK/JNK MAPK pathways, as well as to identify downstream targets of ULK1, to further elucidate the mechanism of action of imeglimin.

The findings of this study reveal novel aspects of imeglimin for clinical applications. Imeglimin is an oral antidiabetic agent used to treat T2DM that can cross the BBB [27]. Therefore, imeglimin has both indirect and direct potential to prevent and/or improve cognitive impairment: (i) the improvement of glucose metabolism could reduce the risk of cognitive impairment by mitigating diabetes-related multifactorial pathologies, and (ii) the stimulation of the ULK1–TXNIP–NLRP3 axis would suppress microglial activation and attenuate neuroinflammation under diabetic conditions. Moreover, imeglimin positively affected HG-induced mitochondrial dysfunction and ROS production, which are implicated in the pathogenesis of neurodegenerative diseases [41]. Accordingly, the pleiotropic actions of imeglimin may exert synergistic beneficial effects on T2DM-associated cognitive impairment. In this respect, imeglimin could contribute to the prognosis of T2DM through good medication adherence by preventing T2DM-associated cognitive impairment. Our findings further suggest the potential application of imeglimin in neurodegenerative diseases such as Alzheimer’s disease, wherein neuroinflammation is implicated in its pathogenesis.

## 5. Conclusions

To our knowledge, this is the first study to demonstrate the novel significance of imeglimin in the suppression of microglial inflammatory responses in an HG environment. Furthermore, the mechanisms underlying the anti-inflammatory effects of imeglimin include the enhancement of the ULK1-mediated suppression of the TXNIP–NLRP3 axis. Future experimental and cohort studies to address the in vivo effects of imeglimin on neuroinflammation under diabetic conditions will be useful in developing innovative strategies to prevent T2DM-associated cognitive impairment.

## Data Availability

The data presented in this study are available from the corresponding authors upon reasonable request.

## Supplementary Materials

The following supporting information can be downloaded at: https://www.mdpi.com/article/10.3390/cells13030284/s1, Figure S1: Cytotoxic effects of imeglimin on BV2 cells; Table S1: List of primer sequences.; Table S2: List of antibodies. References [42,43,44] are cited in Supplementary Materials.
