# Supplementary material for: Imeglimin Exhibits Novel Anti-Inflammatory Effects on High-Glucose-Stimulated Mouse Microglia through ULK1-Mediated Suppression of the TXNIP–NLRP3 Axis
Source: Cells. 2024 Feb 5;13(3):284. doi: 10.3390/cells13030284 (PMC10854746; doi:10.3390/cells13030284)
Supplement: Supplementary file 1 [file cells-13-00284-s001.zip › cells-2772117-supplementary.pdf]

Cells

Supplementary Materials for

Imeglimin Exhibits Novel Anti-Inflammatory Effects on High Glucose-Stimulated Mouse Microglia through ULK1-Mediated Suppression of the TXNIP–NLRP3 Axis

Hisashi Kato, Kaori Iwashita, Masayo Iwasa, Sayaka Kato, Hajime Yamakage, Takayoshi Suganami, Masashi Tanaka and Noriko Satoh-Asahara

This file includes Table S1, Table S2, and Figure S1.

Supplementary Table S1. List of primer sequences.

Genes		Primers (5'-3')	Reference
Il-1β	Forward	CTGAACTCAACTGTGAAATGCCA	[1]
	Reverse	AAAGGTTTGGAAGCAGCCCT	
Tnfa	Forward	ACCCTCACACTCAGATCATCTTC	[2]
	Reverse	TGGTGGTTTGCTACGACGT	
Hmgb1	Forward	CGCGGAGGAAAATCAACTAA	[3]
	Reverse	TCATAACGAGCCTTGTCAGC	
Ulk1	Forward	CACTGCGTGGCTCACCTAAG	[4]
	Reverse	AGCCAACAGGGTCAGCAAAT	
18s	Forward	CGATGCTCTTAGCTGAGTGT	[1]
	Reverse	GGTCCAAGAATTTCACCTCT	

References

1. Iwasa, M.; Kato, H.; Iwashita, K.; Yamakage, H.; Kato, S.; Saito, S.; Ihara, M.; Nishimura, H.; Kawamoto, A.; Suganami, T., et al. Taxifolin Suppresses Inflammatory Responses of High-Glucose-Stimulated Mouse Microglia by Attenuating the TXNIP-NLRP3 Axis. *Nutrients* **2023**, *15*, doi:10.3390/nu15122738.
2. Yang, W.; Zhou, K.; Zhou, Y.; An, Y.; Hu, T.; Lu, J.; Huang, S.; Pei, G. Naringin Dihydrochalcone Ameliorates Cognitive Deficits and Neuropathology in APP/PS1 Transgenic Mice. *Front. Aging Neurosci.* **2018**, *10*, 169, doi:10.3389/fnagi.2018.00169.
3. Lowe, P.P.; Gyongyosi, B.; Satishchandran, A.; Iracheta-Vellve, A.; Cho, Y.; Ambade, A.; Szabo, G. Reduced gut microbiome protects from alcohol-induced neuroinflammation and alters intestinal and brain inflammasome expression. *J. Neuroinflammation* **2018**, *15*, 298, doi:10.1186/s12974-018-1328-9.
4. Goldberg, A.A.; Nkengfac, B.; Sanchez, A.M.J.; Moroz, N.; Qureshi, S.T.; Koromilas, A.E.; Wang, S.; Burelle, Y.; Hussain, S.N.; Kristof, A.S. Regulation of ULK1 Expression and Autophagy by STAT1. *J. Biol. Chem.* **2017**, *292*, 1899-1909, doi:10.1074/jbc.M116.771584.

Supplementary Table S2. List of antibodies.

Antibodies	Supplier	Catalog no.
anti-β-actin	Cell Signaling Technology (CST), Danvers, MA, USA	#4967
anti-AMPKα	CST	#5831
anti-ASC	CST	#67824
anti-ATG7	CST	#8558
anti-Cleaved caspase-1 Asp296	CST	#89332
anti-Cleaved caspase-3 Asp175	CST	#9661
anti-Caspase-3	CST	#9662
anti-GAPDH	CST	#2118
anti-HMGB1	CST	#3935
anti-NLRP3	CST	#15101
anti-Parkin	CST	#2132
anti-PARP	CST	#9532
anti-phospho ULK1 Ser555	CST	#5869
anti-phosho AMPKα Thr172	CST	#2535
anti-TXNIP	CST	#14715
anti-ULK1	CST	#8054
anti-LC3	MEDICAL & BIOLOGICAL LABORATORIES (MBL), Tokyo, Japan	M152-3
anti-p62	MBL	PM045
anti-Caspase-1	Proteintech, Rosemont, IL, USA	22915-1-AP
anti-PINK1	Novus Biologicals, Centennial, CO, USA	BC100-494
anti-mouse IgG, HRP-linked antibody	CST	#7076
anti-rabbit IgG, HRP-linked antibody	CST	#7074

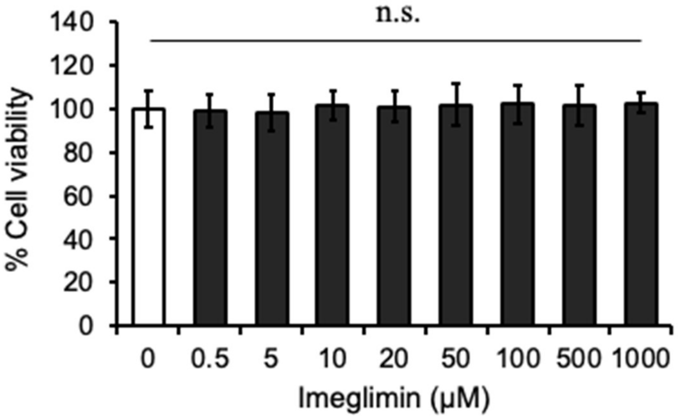

Figure S1. Cytotoxic effects of imeglimin on BV2 cells. Cells were incubated with imeglimin at the dose indicated or the vehicle control for 24 h. The cytotoxicity was determined using a colorimetric assay based on dehydrogenase activity. Data are presented as the mean ± SEM from three independent experiments (*n* = 3). n.s.: not significant, SEM: standard error of the mean.
